# Regulatory Focus, Boundaryless Mindset, and Creativity Among Chinese College Students: A Trait Activation Perspective

**DOI:** 10.3389/fpsyg.2021.670394

**Published:** 2021-09-14

**Authors:** Nan Luo, Xun Xin, Haihong Li, Xuan Yu

**Affiliations:** ^1^School of Business Administration, Chongqing Technology and Business University, Chongqing, China; ^2^Business School, Southwest University of Political Science and Law, Chongqing, China

**Keywords:** regulatory focus, boundaryless mindset, creativity, stressful life events, college students

## Abstract

We explored the impact of two types of regulatory focus on creativity among 330 college students in China, along with the mediating role of boundaryless mindset and moderating role of stressful life events. A three-wave survey showed that both promotion focus and prevention focus positively predicted the creativity in college students, but the positive effect of promotion focus on the creativity in college students was greater than that of prevention focus; boundaryless mindset mediated the relationship between regulatory focus and creativity; stressful life events moderated the direct effect that promotion focus has on boundaryless mindset, and it also moderated the indirect effect that promotion focus has on creativity *via* boundaryless mindset. These results extend the existing research on creativity and establish a new mediating mechanism and boundary conditions between regulatory focus and creativity in college students. Finally, we hope to provide a reference for innovation education.

## Introduction

With the transformation and upgradation of industries, the Chinese government has implemented an innovation-driven development strategy. As an ability to generate novel and useful ideas (Shalley et al., 2004), creativity is closely related to innovation (Gurteen, 1998). As college students play a key role in the development of a country, we should think about stimulating and cultivating their creativity, especially in higher education (Wu et al., 2014). Studies have pointed out that regulatory focus is a significant antecedent of creativity (e.g., Lam and Chiu, 2002). Previous studies have explored the possible mediation mechanism that may exist between regulatory focus and creativity, such as the motivation of desired goal attainment (Förster et al., 2005), improvement of cognitive functions (Friedman and Förster, 2001), happy/sad emotional state (Baas et al., 2011), and inclusive and flexible ways of thinking (Beuk and Basadur, 2016). However, most of the existing studies on the mediators between regulatory focus and creativity are from the individual perspective, while ignoring that the environment is also one of the critical elements of creativity (Sternberg and Lubart, 1996). Therefore, it is essential for us to study the mediating mechanism from the perspective of the interaction between the individual and the environment.

Boundaryless mindset, an individual's overall attitude toward cooperating across boundaries (Briscoe et al., 2006), can be seen as a bridge between the individual and the environment. Boundaryless mindset implies the possibility of an individual to positively develop relationships across boundaries to create an environment of knowledge heterogeneity (Briscoe et al., 2012), based on which college students can effectively enhance their creativity by communicating with people of varied knowledge and experience across different majors or departments (Del Giudice et al., 2014). Besides, combining the distal–proximal framework of motivation (Kanfer, 1990), it can be known that regulatory focus as a distal long-term trait may affect creativity in college students through their boundaryless mindset (i.e., a proximal construct).

However, even college students with the same or similar individual traits will have different behaviors due to different environments (Lazuras et al., 2009). As we all know, college students are in the transition period of their life, encountering stressful life events (Zuo et al., 2020). According to trait activation theory, situations related to traits may play a moderating role in trait–behavior relations (Tett and Guterman, 2000). Since studies have pointed out that stressful life events can affect the self-regulation process of individuals (McLaughlin and Hatzenbuehler, 2009), we speculate that stressful life events are situations related to regulation focus, which will moderate the effect of regulation focus on boundaryless mindset and creativity in college students.

Based on the above analysis, this research attempts to identify the impact of two different regulatory focuses on creativity in college students with the mediating role of boundaryless mindset and moderating role of stressful life events, expected to provide a reference for universities to carry out personalized innovation education for students.

### Regulatory Focus and Creativity

According to the regulatory focus theory, an individual's self-regulation system is distinguished from promotion focus and prevention focus, leading to differences between the individual's thinking style and behavioral strategies (Crowe and Higgins, 1997). For example, promotion focus makes people willingly take relatively greater risks and actively seek more methods, which are likely to contribute to the improvement of creativity (Higgins, 1998). In contrast, prevention focus makes people tend to avoid undesirable ends by avoidance strategy and have a weak tolerance for risks, which means individuals with prevention focus may not be likely to explore new things (Higgins et al., 2001). So, in the early stage of the regulation focus theory, Higgins (1997) believed that promotion focus might enhance creative thinking in individuals, while prevention focus might weaken creative thinking in individuals.

As the understanding of the regulatory focus has grown, it has been found that prevention focus does not necessarily weaken creativity. This is because creativity is not just about cognitive flexibility but also about cognitive perseverance and persistence, and prevention focus can trigger cognitive perseverance (De Dreu et al., 2008). A study showed that individuals with prevention focus show perseverance in creative projects when faced with unforeseen obstacles, which has a positive impact on creativity (Lam and Chiu, 2002). Thus, both types of regulatory focus have positive effects on creativity in college students.

However, promotion focus allows individuals to own a broader attentional scope and facilitates more conceptual access to mental representations. This implies that promotion focus can stimulate creative insight and divergent thinking when compared to prevention focus (Friedman and Förster, 2001). Furthermore, individuals with promotion focus are more associated with positive mood states (e.g., happiness) than individuals with prevention focus, and creativity is more likely to be enhanced when individuals are in a positive emotional state (Baas et al., 2008). Thus, we think promotion focus has a greater positive effect on the creativity in college students. Based on the above analysis, we proposed the following assumptions:

**Hypothesis 1:** Both promotion focus and prevention focus positively predict creativity in college students, and the positive effect of promotion focus is greater than that of prevention focus.

### Mediating Role of Boundaryless Mindset

Individuals with a boundaryless mindset enjoy cooperating with people outside their department or organization, but remain within the current department or organization (Briscoe and Hall, 2006). This mindset can also apply to college students as they naturally affiliate themselves with a particular major or department. The pursuit of gain increases the risk tolerance for students with promotion focus (Förster et al., 2003), and they will be willing to step out of their comfort zone, even if they are likely to encounter all kinds of unexpected situations while cooperating with people outside their major or department. In the case of students with prevention focus, safety needs make them focus on basic responsibilities (Higgins et al., 2001). In the boundaryless time, tasks across departments become a common phenomenon in work or study (Tolbert, 1996), so students with prevention focus will also be willing to cooperate across boundaries in order to accomplish the tasks. In other words, both types of regulatory focus can influence boundaryless mindset in college students.

As boundaryless mindset has the characteristics of openness and self-determination (Segers et al., 2008), we will pay attention to its influence on individual growth. From the individual perspective, boundaryless mindset can enhance an individual's openness to experience (Rastgar et al., 2014), and people with openness to experience are open to new ideas and like to explore new things and environments (Costa and McCrae, 1992). In fact, openness to experience is highly correlated with creativity (McCrae, 1987). From the environmental perspective, cross-organization or departmental cooperation can be regarded as a low-density social network, which usually means more exposure to new information, diversity, and opportunities (Burt, 2005), and college students with a boundaryless mindset can communicate with students majoring in different majors and establish low-density social networks to obtain knowledge and expertise of different fields, which in turn promotes creativity in students (Huang and Liu, 2015).

Moreover, based on the distal–proximal framework of motivation (Kanfer, 1990), trait regulatory focus, as a distal variable, can influence the boundaryless mindset, a proximal variable, through the process of self-regulation, thus exerting an effect on individual creativity. Previous studies have suggested that promotion focus leads people to experience more support from the low-density network (Zou et al., 2015). Therefore, promotion focus leads individuals to develop a higher level of boundaryless mindset, bringing more creativity to individuals through openness to experience (Rastgar et al., 2014) and knowledge heterogeneity (Burt, 2005). On the other hand, prevention focus makes people enjoy a relatively small range of interpersonal relationships (Zou et al., 2015). In other words, prevention focus has a smaller positive effect on creativity through boundaryless mindset. Therefore, we propose the following assumption:

**Hypothesis 2:** Boundaryless mindset mediates the relationship between regulatory focus and creativity.

### Moderating Role of Stressful Life Events

Since individuals are in a stressful environment, the differences in coping strategies and self-regulation ability are most obvious (Scholer and Higgins, 2010). We assume that stressful life events are situations that are relevant to regulation focus. Following the logic of trait activation theory (Tett and Guterman, 2000), stressful life events will be moderators in the process of trait conversion into behavior.

Expectations, needs, or beliefs based on past experiences affect an individual's perception of events (Lazarus and Folkman, 1984). When there are more stressful life events, because of the need for growth (Crowe and Higgins, 1997), individuals with promotion focus will actively seek to cooperate with others to pursue success as they are more in line with cooperative goals (Bittner and Heidemeier, 2013). Thus, they are more likely to produce a boundaryless mindset. When there are fewer stressful life events, the requirements from the environment are reduced, and the existing resources are sufficient for people to respond (Lazarus and Folkman, 1984), which weakens the positive effect of promotion focus on boundaryless mindset. By contrast, when there are more stressful life events, it becomes potentially risky for individuals with prevention focus to cooperate with others across the department due to the need for safety (Crowe and Higgins, 1997), anxiety and alertness increases among the individuals, and the preferred strategic direction of their regulatory system is not to pose a threat (Higgins and Molden, 2003). Thus, their motivation to build a relationship will be less; when there are fewer stressful life events, they are in a relaxed experience (Higgins et al., 1997), and they are more likely to cooperate with other departments. Therefore, we propose the following:

**Hypothesis 3:** Stressful life events positively moderate the direct effect that promotion focus has on boundaryless mindset (H3a), while they negatively moderate the direct effect that prevention focus has on boundaryless mindset (H3b).

Besides, for individuals with promotion focus, when there are more stressful life events, they develop higher levels of boundaryless mindset as they are more willing to cooperate with others (Bittner and Heidemeier, 2013). This in turn develops higher levels of creativity as they show more openness to experience (Rastgar et al., 2014) and exposure to knowledge heterogeneity (Burt, 2005); when there are less stressful life events, the reverse is true. In the case of individuals with prevention focus, cognitive persistence allows them to persist in difficult tasks (Lam and Chiu, 2002), but when the level of stressful life events is high, according to the stress–vulnerability model (Galvin, 2004), their cognitive resources become impaired, so the positive effect of prevention focus on creativity becomes weakened. When the level of stressful life events is low, their cognitive resources play a protective role to help them develop (Li et al., 2012), and the positive effect of prevention focus on creativity may be enhanced. Thus, based on the hypothetical relationship described above, we propose the following:

**Hypothesis 4:** Stressful life events positively moderate the indirect effect that promotion focus has on creativity *via* boundaryless mindset (H4a), and negatively moderate the indirect effect that prevention focus has on creativity *via* boundaryless mindset (H4b).

**Figure 1:**
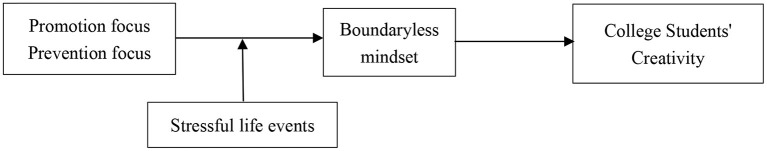
The proposed moderated mediation model.

The moderated mediation model is shown in Figure 1.

## Materials and Methods

### Participants and Procedures

A cluster random sampling method was used to select students from a college in Chongqing, and a total of 330 questionnaires were obtained from them. The above variables (i.e., regulatory focus, boundaryless mindset, creativity and stressful life events) were measured in turn over three weeks. At the first time point (T1), data related to personal information was collected, and the two different regulatory focuses were measured in turn. A week later (T2), stressful life events and boundaryless mindset were measured. Finally, creativity in college students was measured in the third week (T3). Additionally, this research included the last four digits of the mobile phone number, so that the data corresponding to the above variables could be effectively matched, and SPSS 21.0 and Mplus 7.0 were used to analyze and process the data.

A total of 310 completed questionnaires were obtained by matching the last four digits of the mobile phone number, and after excluding the incomplete questionnaires there were 302 (91.50%) valid responses. The characteristics of the sample data were as follows: in terms of gender, 21.5% were men and 78.5% were women; in terms of age, 0.7% were aged 18 years and below, 89.4% were aged between 19 and 21 years, and 9.9% were aged between 22 and 24 years; in terms of ethnic groups, people belonging to Han nationality accounted for 92.5%, while other ethnic minorities accounted for 7.5%.

### Measures

In this study, we used the Chinese version of the stressful life events scale, and the rest of the variables were measured with a mature Western scale. For ensuring the consistency and applicability of the English scale in the Chinese context, the author conducted a translation–back translation procedure (Brislin, 1986). Before the formal investigation, a preliminary test was conducted on 15 college students, and the items were modified based on their feedback. The variables were measured with a 5-point Likert scale ranging from 1 = strongly disagree to 5 = strongly agree, except for creativity in college students.

### Regulatory Focus

Regulatory focus was measured with an 18-item scale developed by Neubert et al. (2008), which includes two dimensions, namely promotion focus (e.g., “I tend to take risks at learning in order to achieve success”) and prevention focus (e.g., “Fulfilling my work duties is very important to me”). In order to make the items in the scale to be more relatable with the actual situation of college students, this study replaced “work” with “learning” and “organization” with “school.” Cronbach's alpha for promotion focus subscale and prevention focus subscale were 0.82 and 0.76, respectively.

### Boundaryless Mindset

Boundaryless mindset was measured with an 8-item boundaryless mindset subscale of the Boundaryless Career Attitude Scale developed by Briscoe et al. (2006). A sample item is “I am energized with new experiences and situations.” Cronbach's alpha for this scale was 0.89.

### Creativity in College Students

We adopted the Person-Environment Fit Scale for Creativity (PEFSC) developed by Sen et al. (2014) to assess creativity in college students. There are 14 items in this scale including two dimensions, individual (e.g., “I like to see different point of views”) and environmental (e.g., “There is cultural diversity in my environment”). Cronbach's alpha for this scale was 0.82.

### Stressful Life Events

Stressful life events were measured with a 27-item Adolescent Self-Rating Life Events Checklist (ASLEC) developed by Liu et al. (1997), which includes six dimensions. The dimensions are relationship (e.g., “Being misunderstood”), learning pressure (e.g., “Examination failure”), punishment (e.g., “criticism”), loss (e.g., “Friends or relatives died”), health and adaption (e.g., “Stay away from my family”), and others (e.g., “Hate school”). Items are rated on a 6-point Likert scale, ranging from 0 to 5 (0 = not occur; 1= no influence; 2 = mild influence; 3 = moderate influence; 4 = severe influence; and 5 = very severe influence). Cronbach's alpha for this scale was 0.92.

### Control Variables

Some demographic variables were selected as control variables, such as gender, age, and nationality because all these variables can influence employee creativity (Kaufman, 2006).

## Result

### Discrimination and Common Method Deviation Test

Mplus 7.0 was used to carry out a series of structural equation model tests (see Table 1 for the results) to examine the distinctions between promotion focus, prevention focus, boundaryless mindset, stressful life events, and creativity in the five-factor model. The results of the confirmatory factor analysis show that the five-factor model fits the actual data well. The specific results are: χ*2(1700)* = 3692.83, TLI = 0.90, CFI = 0.90, RMSEA = 0.06, and SRMR = 0.07, indicating that promotion focus, prevention focus, boundaryless mindset, stressful life events, and creativity represent five different constructs. Although the data were collected at different time points in this study, considering that the questionnaires were answered by college students themselves, common method deviations may exist to some extent. The method of potential factor control was used to test the common method deviation of the model. After adding the common method factor, there was no significant improvement in the simulated fitting index in comparison with the five-factor model [χ*2(1641)* = 2965.09, TLI = 0.91, CFI = 0.91, RMSEA = 0.07, SRMR = 0.06]. Therefore, the common method deviation was not considered important in this study.

**Table 1:** Means, standard deviations, correlation coefficients between variables and reliability coefficients.

**Variables**	**Mean**	**SD**	**1**	**2**	**3**	**4**	**5**	**6**	**7**	**8**
1. Gender	0.79	0.42								
2. Age	2.09	0.31	0.09							
3. Nation	1.13	0.37	0.03	0.15^*^	–					
4. Promotion focus	3.47	0.56	−0 −0.08	0.02	0.09	**0.82**				
5. Prevention focus	3.83	0.45	0.03	−0.05	−0.02	0.10	**0.76**			
6. Boundaryless mindset	3.67	0.69	0.04	0.01	0.05	0.42^***^	0.17^**^	**0.89**		
7. Stressful life events	1.96	0.68	0.03	−0.01	−0.06	−0.05	0.09	−0.03	**0.92**	
8. Creativity	3.76	0.56	0.05	−0.01	0.03	0.38^***^	0.19^***^	0.45^***^	−0.08	**0.82**

*Note: a. N = 302*,

* *p < 0.05*,

** *p < 0.01;*

*** *p < 0.001. The bold values indicates reliability coefficient*.

### Descriptive Statistics

Data characteristic analysis of control variables and major variables, including mean, standard deviation, correlation coefficient, and consistency coefficient of major variables, are shown in Table 1.

### Examining the Mediation Model

Using the structural equation model analysis method, a complete mediation model and a partial mediation model of promotion focus and prevention focus affecting the creativity of college students through boundaryless mindset were constructed. It was found that the partial mediation model was well-fitted, and all the fit indexes were in line with the fitness standard *(*χ*2* = 3692.83, TLI = 0.91, CFI = 0.91, RMSEA = 0.05, SRMR = 0.07), while the fit indexes of the complete mediation model were poor, and the indexes of fitting goodness were significantly worse than those of partial mediation models [Δχ*2 (2)* = 21, *p* < 0.05]. Therefore, the partial mediation model was retained in this study to verify the research hypothesis.

**Figure 2:**
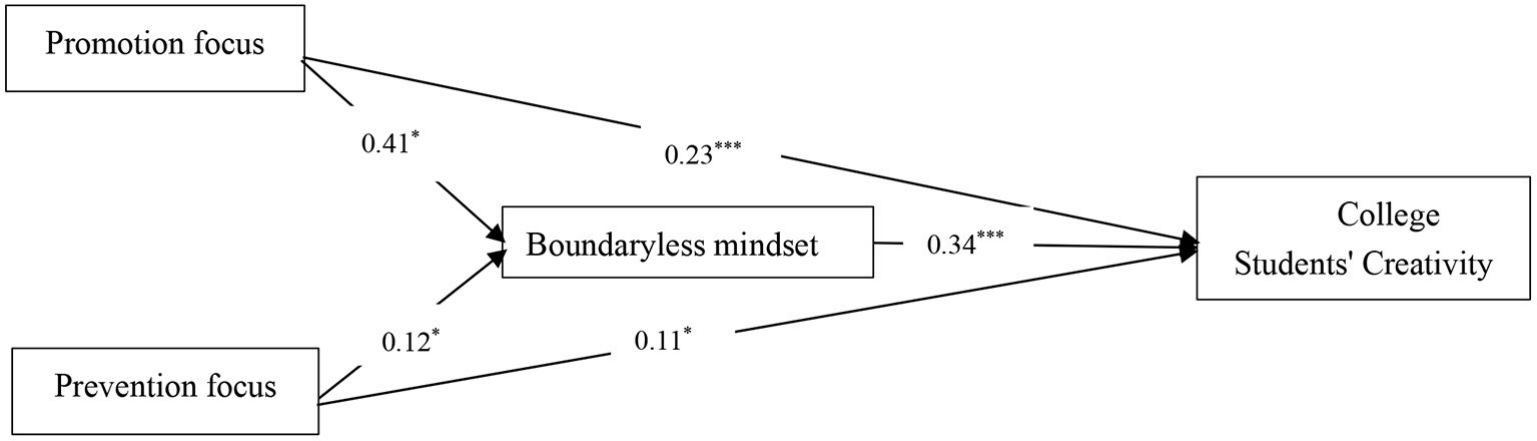
The partial mediation model where promotion focus and prevention focus through the boundaryless mindset affect creativity.

The standardized regression coefficients of each direct path are shown in Figure 2. According to Figure 2, promotion focus directly positively affects creativity in college students (β = 0.23, *p* < 0.001), and similarly, prevention focus also directly positively affects creativity in college students (β = 0.11, *p* < 0.001), but the positive effect of promotion focus on creativity in college students was greater than that of prevention focus. Hence, Hypothesis H1 is verified.

For indirect effects, both types of regulatory focus were found to positively affect creativity *via* the boundaryless mindset. To ensure the consistency and stability of the results of indirect effect analysis, the error correction method of 2000 Bootstrap samples was adopted to obtain the estimated point value and estimated interval of the indirect effect, as shown in Table 2. According to Table 2, the mediating effect of boundaryless mindset between promotion focus and creativity is β = 0.134, and the confidence interval at the 99% level does not contain 0; the mediating effect of boundaryless mindset between prevention focus and creativity is 0.052, and the confidence interval at the 95% level does not contain 0. It can be seen that boundaryless mindset mediates the relationship between regulatory focus and creativity. Assume that H2 is verified.

**Table 2:** Mediating effect value of boundaryless mindset.

**Independent variable**	**Point estimation**	**95% confidence interval**		**99% confidence interval**	
		**Upper limit**	**Lower limit**	**Upper limit**	**Lower limit**
Promotion focus	0.134	0.076	0.192	0.058	0.210
Prevention focus	0.052	0.002	0.102	−0.013	0.118

### Testing the Moderated Mediation Model With Stressful Life Events as Moderator

#### Stressful Life Events Moderate the Relationship Between Promotion Focus and Creativity *via* Boundaryless Mindset

**Figure 3:**
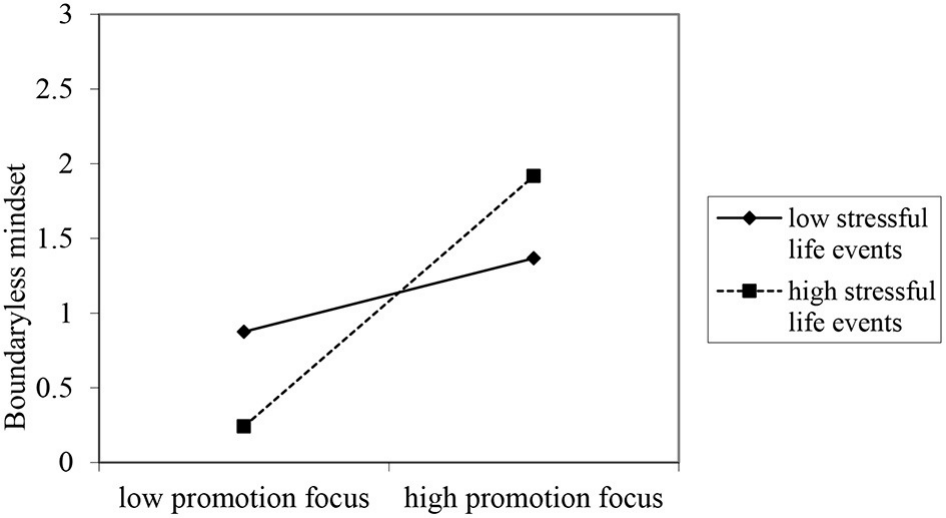
The moderating effect of stressful life events on promotion focus to boundaryless mindset.

Mplus 7.0 was used to test whether stressful life events moderate the indirect effect of promotion focus on creativity through boundaryless mindset. Statistically, stressful life events positively moderated the relationship between promotion focus and boundaryless mindset, as shown in Figure 3. Assume that H3a is verified.

**Figure 4:**
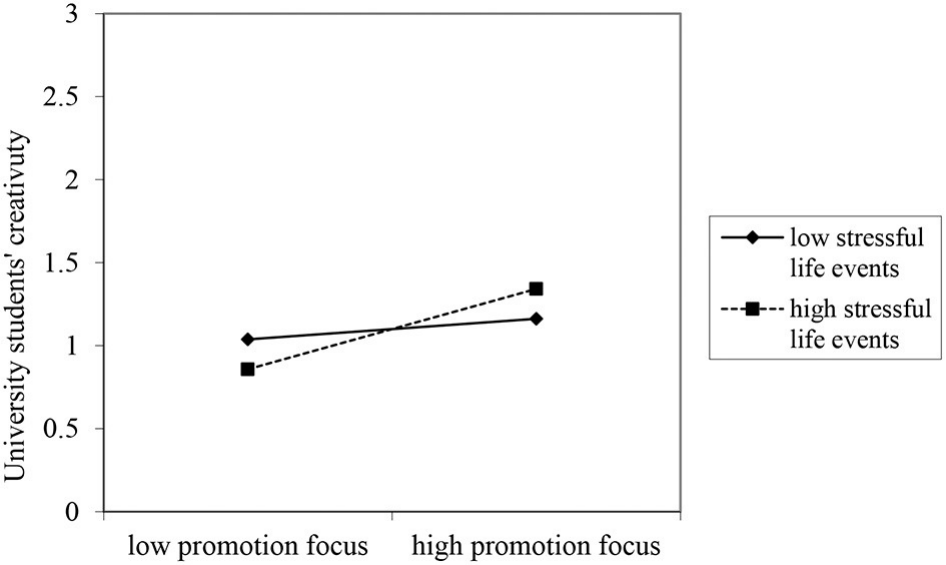
Moderated mediating effects of stressful life events.

Next, we tested the effect values of the first and second phases, as well as the direct and indirect effects when stressful life events was positive and negative by one standard deviation (see Table 3). As shown in Table 3, stressful life events do not significantly moderate the direct effect of promotion focus on creativity, but the indirect effect of promotion focus on creativity through boundaryless mindset (β = 0.115, *p* < 0.05) (see Figure 4). Bootstrap2000 parametric autonomous sampling was done to test the stability of the moderated mediating effect. The analysis results showed that the difference between the indirect effects of boundaryless mindset under high and low levels of stressful life events was still significant, with a confidence level of 95% and a confidence interval of [0.028, 0.248]. Therefore, Hypothesis H4a is verified.

**Table 3:** Simple effect analysis of moderated mediating effects of stressful life events (X = promotion focus).

**Moderator variables**	**Phase**			**Effect**		
	**1**	**2**		**Direct**	**Indirect**	**Overall**
**Stressful life events**						
high	0.746^***^	0.280^***^	0.221^*^	0.209^***^	0.430^***^	
low	0.338^***^	0.280^***^	0.220^***^	0.095^*^	0.315^***^	
difference	0.409^*^	0.000	0.001	0.115^*^	0.115^*^	

*The standard deviations of stressful life events are 0.90;*

*** *p < 0.001*,

* *p < 0.5*.

#### Stressful Life Events Moderate the Relationship Between Prevention Focus and Creativity *via* Boundaryless Mindset

Mplus 7.0 was used to test whether the stressful life events moderated the indirect effect that prevention focus had on creativity through boundaryless mindset. First, it was found from the statistical results that the moderating effect of stressful life events between prevention focus and boundaryless mindset was only marginally significant (β = 0.273, *P* = 0.093 < 0.1). Based on the stability test of Bootstrap2000 parametric autonomous sampling, it was found that the confidence interval at 95% was [−0.067, 0.577] and the interval contained 0, indicating that the moderating effect of stressful life events between prevention focus and boundaryless mindset was not significant at the 95% level. At the same time, simple slope analysis also found that prevention focus had a significant positive effect on boundaryless mindset at a high level of stressful life events *(*β = 0.468, *P* ≤ 0.01); while the level of stressful life events were low, prevention focus had no effect on boundaryless mindset. However, the simple slope difference was not significant, i.e., the moderating effect was not significant. H3b is not verified.

In the analysis of the moderating mediation effect, since the basic condition that the moderating variable should moderate the relationship of the independent variable with the mediator is not satisfied, this article does not list the moderating mediation result. H4b is not verified.

## Discussion

Based on the distal–proximal framework of motivation, this study explores the effects of regulatory focus on creativity in college students through boundaryless mindset and examines the effect of stressful life events as boundary conditions.

### Theoretical Implications

Firstly, our study found that both types of regulation focus can positively predict creativity in college students, but the positive effect of promotion focus is greater than that of prevention focus. This result is different from the results of previous studies (e.g., Förster and Dannenberg, 2010), which believe that prevention focus weakens creativity. As we discussed before, the positive influence of prevention focus on creativity can be understood from cognitive perseverance and mood state, and we cannot simply think prevention focus is a negative trait; combined with different task settings (Lam and Chiu, 2002) and emotional experiences (De Dreu et al., 2008), we can realize more about the mechanism of prevention focus.

Secondly, boundaryless mindset is a mediating mechanism between regulation focus and creativity in college students. The verification of Hypothesis 2 shows that both types of regulatory focus can positively influence the creativity in college students *via* boundaryless mindset. Existing studies explore the outcomes of boundaryless mindset, concentrated mainly in the field of career development (e.g., vocational adaptability, Stumpf, 2014; organizational commitment, Briscoe Jon and Finkelstein Lisa, 2009). This study tries to extend the application of boundaryless mindset to the field of individual growth and development, and the gains obtained will be helpful in providing a reference for subsequent studies. Consistent with the underlying logic of previous literature (Fernandez and Enache, 2008; Guan et al., 2019), as an adaptive mindset, boundaryless mindset can have a strong positive influence on individuals in providing them with broad thinking and vision.

The results show that stressful life events positively moderate the direct effect of promotion focus on boundaryless mindset, and positively moderate the indirect effect that promotion focus has on creativity *via* boundaryless mindset. However, the moderating effect of stressful life events between prevention focus and boundaryless mindset is not significant, implying that Hypotheses 3B and 4B are not valid, which may be explained from the mood activation perspective. In spite of the high levels of stressful life events, prevention focus may increase anxiety and alertness, making individuals reluctant to establish relationship and cooperation across the boundary, because they fear that their inputs (such as time and energy) may be lesser than their gains in this process. However, individuals with prevention focus may also get activated by avoiding undesirable end states (Carver, 2004), and with a moderate level of arousal, they are motivated to search for information and consider multiple options (De Dreu et al., 2008). In this case, individuals with prevention focus also actively establish relationships across boundaries to cope with the current situation. Thus, it may be that these two opposing mechanisms contribute to the result that stressful life events do not have a moderating effect between prevention focus and boundaryless mindset.

### Practical Implications

These results are expected to provide a reference for educators and students.

First, in college education, institutions should carry out training activities on the basis of distinguishing the different regulatory focuses of individual students. We only need to give them more autonomy and provide them with the corresponding guidance. In case of students with prevention focus, as the regulatory focus could be guided by a real-time situation (Higgins, 1997), we can also induce their situational promotion focus by setting up a reasonable task frame to activate their pursuit of success, for example, provide feedback on student performance using descriptive words instead of critical words.

Second, existing studies have shown that the mindset of students can be changed (Yeager and Dweck, 2012). Thus, mindset intervention measures are necessary for students with prevention focus. In the class, the teacher should consciously stimulate the growth of boundaryless mindset among students, such as arranging the relevant simulation training and assigning tasks that involve cooperation with others.

Third, since students with promotion focus often perform better in the face of stressful life events, we should pay more attention to students with prevention focus. Studies have shown that individuals experience threats when situational needs are perceived to exceed resources (Lazarus and Folkman, 1984; Blascovich and Tomaka, 1996), so the most important thing for students with prevention focus is to accumulate as much capital as possible, both psychological and social, to cope better with stress. College counselors can often communicate with students to help them build good interpersonal relationships and provide them with support and encouragement. Stress management education is also essential, and universities should regularly hold psychology lectures to teach students to correctly realize stress and actively deal with it.

### Limitations and Future Directions

We believe that this study can be improved through the following points:

First, we applied regulatory focus as a long-term personality trait, but some studies have pointed out that regulatory focus can be temporarily induced in a specific situation (Baas et al., 2011). Therefore, in future studies, we can combine experimental methods to manipulate the regulatory focus of individuals through tasks or language frameworks.

Second, in this study, we used the subjective self-report questionnaire to measure creativity; however, the initial creative ideas and the final creative results are both different stages of creativity (Caniëls et al., 2014), so we should also measure creativity in college students combined with objective indicators, such as invention patents, published research, and innovation papers.

Finally, there are 67 items to measure variables, according to the principle of the linear regression analysis that the number of valid questionnaires no less than five times the number of items (i.e., at least 335 questionnaires) (Hair et al., 1998). In this paper, the effective sample size has not reached the ideal effective sample size, and women account for 78.5%, which may influence the conclusion. Therefore, we can continue to expand the sample size and increase the number of samples by including more men to improve the external validity of the study.

## Data Availability Statement

The original contributions presented in the study are included in the article/supplementary material, further inquiries can be directed to the corresponding author/s.

## Ethics Statement

Written informed consent was obtained from the individual(s) for the publication of any potentially identifiable images or data included in this article.

## Author Contributions

NL wrote the manuscript and analyzed the data under the guidance of XX and XY. XX contributed to data analysis and editing of the manuscript. HL contributed to study design and data collection. XY contributed to study design and critical revisions. All authors contributed to the article and approved the submitted version.

## Funding

This study was supported by the Youth Project of the National Natural Science Foundation of China (71802033), the National Natural Science Foundation of China (71902166), the China Postdoctoral Science Foundation (2018M643786XB), and supported by the 67th batch of China Postdoctoral Science Foundation (2020M673191).

## Conflict of Interest

The authors declare that the research was conducted in the absence of any commercial or financial relationships that could be construed as a potential conflict of interest.

## Publisher's Note

All claims expressed in this article are solely those of the authors and do not necessarily represent those of their affiliated organizations, or those of the publisher, the editors and the reviewers. Any product that may be evaluated in this article, or claim that may be made by its manufacturer, is not guaranteed or endorsed by the publisher.
